# Neural and Behavioral Evidence for an Intrinsic Cost of Self-Control

**DOI:** 10.1371/journal.pone.0072626

**Published:** 2013-08-27

**Authors:** Wouter Kool, Joseph T. McGuire, Gary J. Wang, Matthew M. Botvinick

**Affiliations:** 1 Department of Psychology, Princeton University, Princeton, New Jersey, United States of America; 2 Princeton Neuroscience Institute, Princeton University, Princeton, New Jersey, United States of America; 3 Department of Psychology, University of Pennsylvania, Philadelphia, Pennsylvania, United States of America; Georgia State University, United States of America

## Abstract

The capacity for self-control is critical to adaptive functioning, yet our knowledge of the underlying processes and mechanisms is presently only inchoate. Theoretical work in economics has suggested a model of self-control centering on two key assumptions: (1) a division within the decision-maker between two ‘selves’ with differing preferences; (2) the idea that self-control is intrinsically costly. Neuroscience has recently generated findings supporting the ‘dual-self’ assumption. The idea of self-control costs, in contrast, has remained speculative. We report the first independent evidence for self-control costs. Through a neuroimaging meta-analysis, we establish an anatomical link between self-control and the registration of cognitive effort costs. This link predicts that individuals who strongly avoid cognitive demand should also display poor self-control. To test this, we conducted a behavioral experiment leveraging a measure of demand avoidance along with two measures of self-control. The results obtained provide clear support for the idea of self-control costs.

## Introduction

Human decision-makers enjoy an important, though fallible, capacity for *self-control*: an ability to resist immediate pleasures in favor of longer-term goals. The importance of this faculty, and the consequences of its occasional failure, are evident from everyday life. Scientific investigation has linked individual differences in self-control to significant life outcomes, including obesity, academic performance, and mental health [1]–[3]. As such findings have emerged, the goal of understanding the principles and mechanisms underlying self-control has come increasingly to the fore.

**Figure 1:**
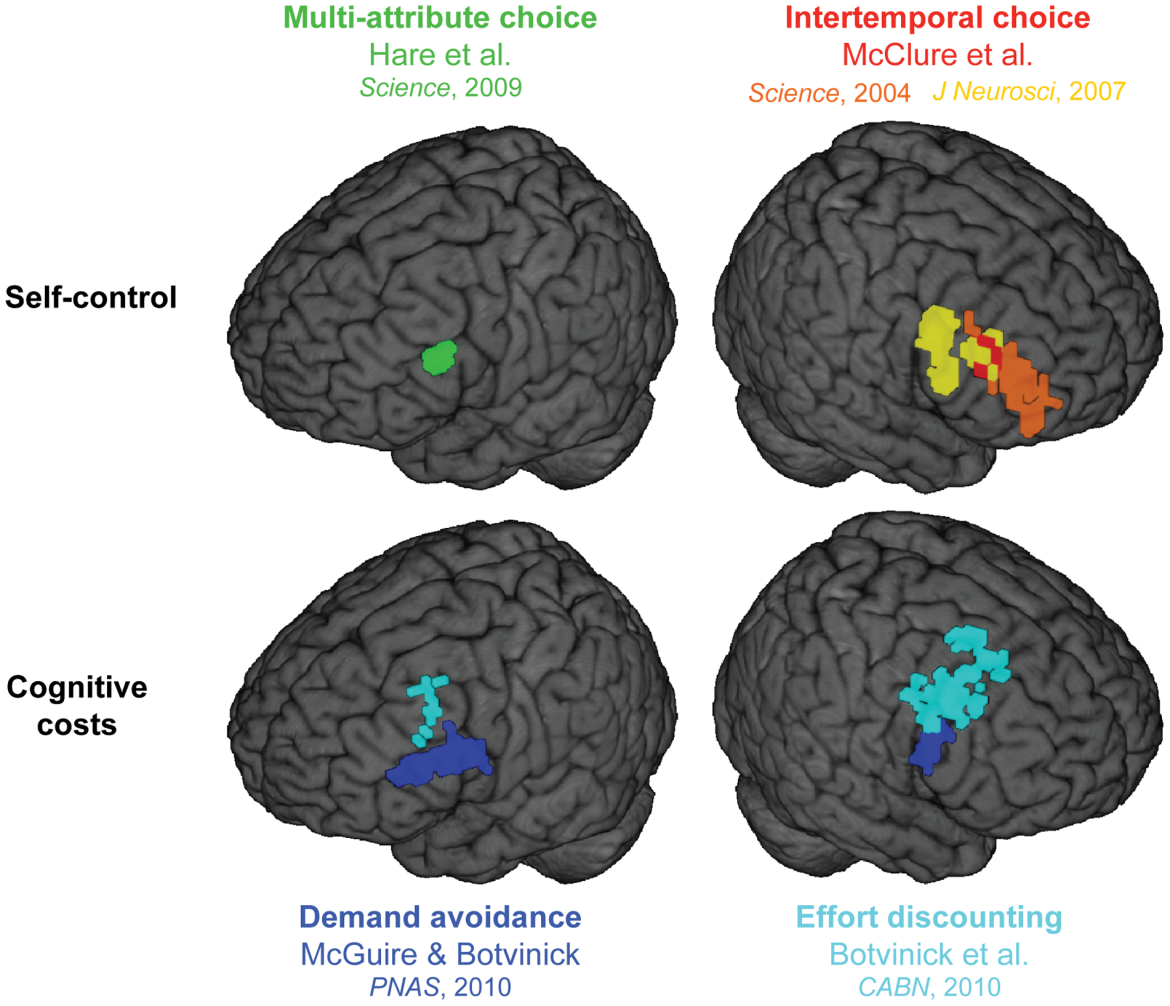
dlPFC regions identified in five neuroimaging studies. The upper tier shows areas displaying effects related to self-control in multi-attribute choice [8] and ITC [9], [10]. The lower tier shows areas displaying effort cost effects (demand avoidance [21] and effort discounting [22]). The images were rendered in three-dimensional space using AFNI’s ‘Render dataset’ function [36].

Over recent years, behavioral economics has generated several formal theoretical models of self-control. In what is now arguably the modal model [4]–[7], the exertion of self-control involves the overriding of a ‘short-term self,’ fixated on immediate rewards, by a ‘long-term self,’ which seeks to maximize reward over the long-term. Apparent support for this dual-self view has recently come from a set of neuroscientific studies focusing on dorsolateral prefrontal cortex (dlPFC). In one such study, Hare, Camerer and Rangel [8] used functional magnetic resonance imaging (fMRI) to measure brain activity as dieters performed a multi-attribute choice task involving foods varying in tastiness and healthfulness. When participants displayed self-control, making choices consistent with their diets, fMRI revealed accompanying activation in dlPFC (Figure 1), activity that was reduced when participants chose more impulsively. A similar finding has come from studies of *intertemporal choice* (ITC), where decisions are made between tempting immediate rewards and larger delayed rewards. In two studies of ITC, McClure and colleagues [9], [10] observed increased activity within a network including the dlPFC, concurrent with selection of the delayed option^1^ (see Figure 1) – behavior understood to reflect self-control [11], [12]. Transient inactivation of dlPFC has subsequently been shown to yield more impatient behavior in ITC [13].

These recent studies have given rise to competing views on the precise role of dlPFC in self-control^2^ and the data are not free of inconsistency [14], [15]. Nevertheless, taken together, the available findings do appear to provide support for the dual-self view, by supplying evidence for an isolable neural system whose activity produces patient, far-sighted choices, and whose inactivity releases more impulsive behavior [16].

Such evidence speaks to the most salient assumption of the prevailing economic framework, the idea of dual ‘selves.’ However the economic model also depends upon a second key assumption: It supposes that self-control is *costly.* The exertion of self-control is assumed, within the dual-self framework, to carry inherent disutility [17]. For self-control to be imposed, its expected payoffs must surpass this intrinsic cost [4]–[7].

The cost of self-control plays a pivotal role in the dual-self framework, empowering it to account for a range of important behavioral phenomena [5]–[7]. However, in contrast to the assumption of dual selves, no independent experimental evidence has yet been brought to bear on this tenet of the standard model. Despite its theoretical appeal, the notion of self-control costs stands in need of empirical validation.

**Figure 2:**
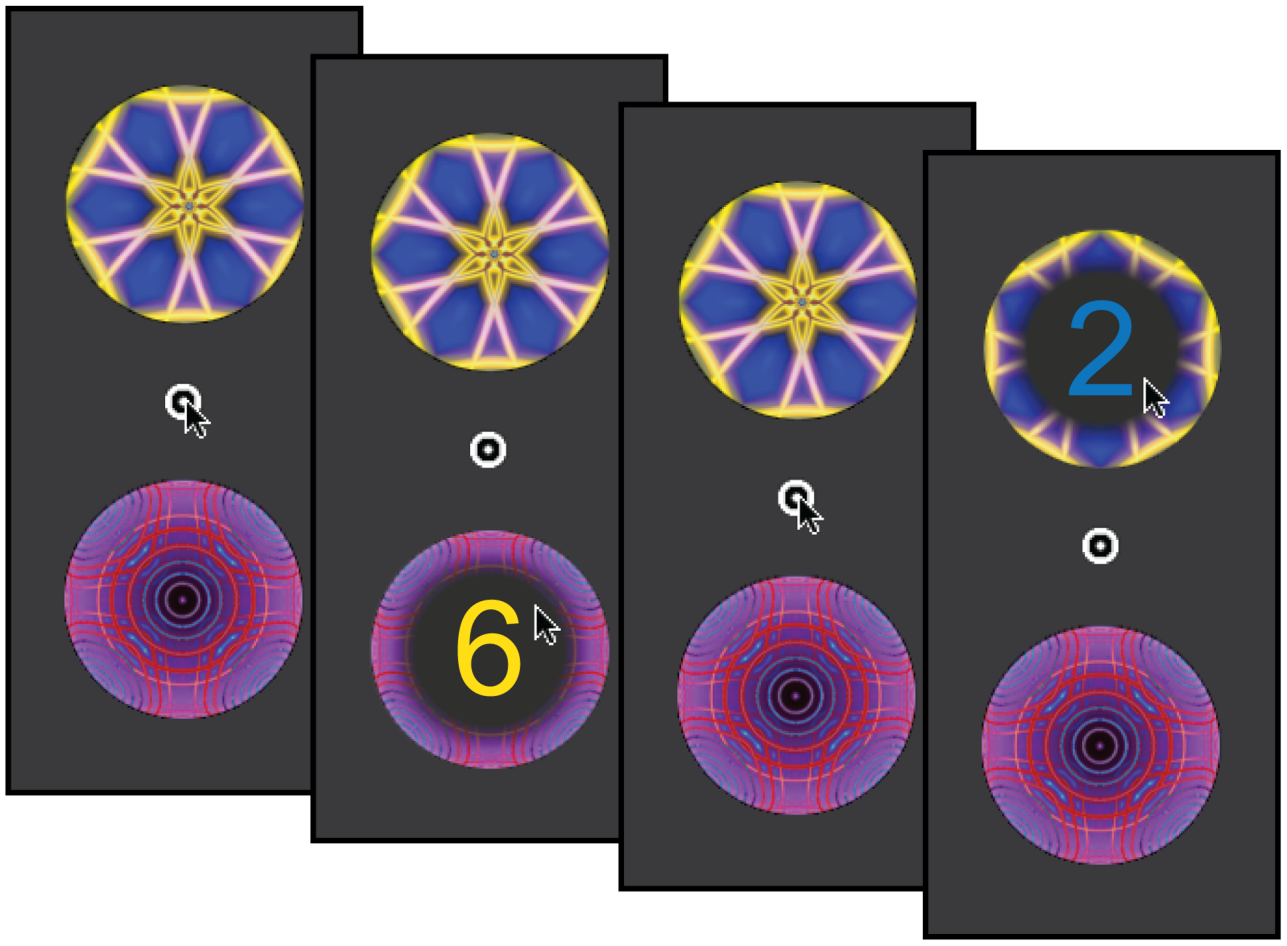
Sample event sequence from the DST. On each trial, the participant selects freely between two patterned targets, which vary in appearance and relative position every 75 trials. Selection of a target reveals an Arabic numeral. Depending on the color of the numeral (blue or yellow), the participant uses a key-press to render either a parity (odd/even) judgment or magnitude (less/greater than five) judgment. Unannounced to the participant, one target yields numerals that tend to vary in color across trials (90% of selections), imposing executive demand through the requirement to switch stimulus-response mapping. The other target yields numerals that tend to maintain a consistent color (90% of trials). Demand avoidance is quantified as the proportion of trials on which the low-demand target is selected, across the entire experiment.

With this desideratum in mind, we propose that it may be useful to consider recent findings from a different research domain, where evidence for cost-sensitive decision making has recently emerged. Work on *executive function* investigates the set of capacity-limited mechanisms that coordinate basic information-processing resources, including memory, attention, action selection and other faculties, in the service of specific tasks [18]. A set of recent experiments has provided evidence that demands on executive function register as subjectively costly or aversive. This evidence comes, in part, from work with the *demand selection task* (DST), a behavioral paradigm in which participants choose repeatedly between task options associated with differing levels of executive demand (Figure 2). While there is important variability across individuals, overall behavior in the DST displays a pattern of demand avoidance, a bias away from the choice option that carries greater executive demand [19].

Recent neuroimaging experiments link the demand avoidance effect with neural activity in the dlPFC, a region known to be critical for executive control [20]. McGuire and Botvinick [21], for example, found that the degree to which the dlPFC was recruited during task performance predicted subsequent avoidance of the same task (Figure 2). Data from a related study by Botvinick, Huffstetler and McGuire [22] implicate the dlPFC in cognitive *effort discounting* (see Information S!). Here, dlPFC activity arising during performance of a cognitively demanding task predicted later reductions in striatal responses to rewards presented as payments for the task (Figure 1).

These studies examined the cost of cognitive effort in settings quite different from those involved in the self-control research introduced earlier. However, it seems natural to consider whether the cost of cognitive control, as demonstrated in this research on executive function, might bear a connection with the cost of self-control hypothesized in the economic dual-self model. The existence of such a connection is suggested by the fact that neuroscience research on cognitive costs and separate research on self-control have both converged on the dlPFC. Indeed, the specific areas implicated in the two domains of research bear a striking resemblance to one another as can be seen in Figure 1. In this Figure, we present dlPFC regions identified in five neuroimaging studies. The top of the Figure depicts areas in dlPFC that display effects related to self-control from both the multi-attribute study by Hare and colleagues [8] and the ITC studies by McClure and colleagues [9], [10], both of which are described above. The lower part shows areas displaying effort costs effects in dlPFC from our own lab (demand avoidance [21] and effort discounting [22]).

To test for genuine overlap, we conducted a set of region-of-interest analyses, returning to the fMRI datasets that demonstrated demand avoidance [21] and effort-discounting [22] effects in dlPFC, but testing for these effects within the dlPFC regions identified in the dieter study by Hare and colleagues [8], and the ITC studies by McClure and colleagues [9], [10]. In every case tested, the dlPFC regions from these self-control studies displayed statistically significant effort-cost effects (Table 1; see Information S1).

**Table 1:** Results from a set of region-of-interest analyses. dlPFC regions-of-interest (ROI) were drawn from fMRI studies on self-control in multi-attribute choice and in ITC [8]–[10].

		McClure et al., 2004		McClure et al., 2007		Hare et al., 2009	
		ITC – Monetary rewards		ITC – Primary rewards		Self control – dieter study	
Dataset	Effect	*t* (df)	*p*	*t* (df)	*p*	*t* (df)	*p*
McGuire & Botvinick, 2010	Demand avoidance	1.90 (9)	<0.05	3.03 (9)	<0.01	3.11 (9)	<0.01
Botvinick et al., 2009	Effort discounting	−2.68 (22)	<0.05	−2.99 (22)	<0.005	−1.81 (22)	<0.05

Using data from two previous studies [21], [22], we tested whether task-induced activity in these regions predicted a reduction in reward-receipt responses in ventral striatum, i.e. effort discounting [22], and such activity predicted subsequent task-avoidance behavior [21]. All tests yielded significant effects, based on one-tailed t-tests.

This anatomical intersection between self-control and effort-cost effects suggests a functional connection between these two, consistent with the idea that self-control itself carries intrinsic subjective costs. If this connection is real, then a behavioral prediction follows. The idea relates to individual differences in cost processing and self-control. Specifically, individuals who are particularly sensitive to cognitive costs, as reflected in strong demand avoidance, should display relatively weak self-control.

To test this, we conducted an experiment in which fifty participants each completed three tasks, in counterbalanced order. Each participant’s propensity to avoid cognitive demand was measured using the DST illustrated in Figure 2. Self-control was measured in two ways. First, participants completed the Self-Control Scale [3], a standardized self-report measure involving 36 questions concerning self-regulation in everyday life. Second, participants performed an ITC task closely based on the one used in the fMRI study of McClure and colleagues [10]. Based on previous findings, we anticipated significant variability across participants within each of the three tasks. More importantly, we predicted that the strength of demand avoidance in the DST would correlate inversely with both measures of self-control.

## Materials and Methods

### Participants

Fifty students from the Princeton University (31 females, 18–24 years) participated, providing informed consent and receiving $10 plus whatever bonus was received in the ITC task following procedures approved by the Princeton University Institutional Review Board.

### Procedure

All participants performed an ITC task and the DST, with order counterbalanced across participants, followed by completion of the Self-Control Scale [3]. Both the ITC task and the DST were programmed using Matlab and the Psychophysics Toolbox [23], [24].

In the ITC task, all participants were presented with an identical sequence of 100 unique choice trials. In each trial, participants chose between two monetary offers, one involving a smaller sum to be delivered immediately following the experiment, the other a larger sum to be conferred after a specified delay. To construct the offer sequence, immediate rewards were sampled from a normal distribution with mean $8 and standard deviation of $2 (range $3.01–$12.85). The delayed option was between 5% and 50% larger than the immediate option (range $3.93–$16.69) and was available after a period ranging between two and ten weeks, in one-week increments. Participants were (truthfully) informed that one of their selections, from a randomly chosen trial, would be awarded as an Amazon gift card at the selected time-point.

The DST was drawn without modification from Kool et al. [19]. The task was divided into eight runs of 75 trials, each featuring a visually contrasting pair of choice targets (Figure 2). The position and appearance of the targets remained fixed within each run but varied across runs, always appearing along the perimeter of a virtual circle, and separated by an angular distance of 45 degrees. Participants were told they were free to sample from either target, but that if they developed a preference they should feel free select one more than the other. Selection of a target revealed an Arabic numeral. Depending on the color of the numeral (blue or yellow), the participant used a key-press to render either a parity (odd/even) judgment or magnitude (less/greater than five) judgment. During each run, the numerals in one (*high-demand)* target switched color relative to the previous trial – requiring an effortful stimulus-response remapping – with a probability of 0.9. In the other (*low-demand)* target, colors switched with a probability of 0.1. Participants’ demand-avoidance score was computed as the proportion of trials on which the low-demand cue was selected.

The Self-Control Scale [3] is a 36-item questionnaire that measures self-regulatory behavior throughout four domains: thoughts, emotions, impulses and performance. It comprises a list of statements (e.g., ‘I am always on time’), whose self-relevance participants rate using a five-point scale.

## Results

**Figure 3:**
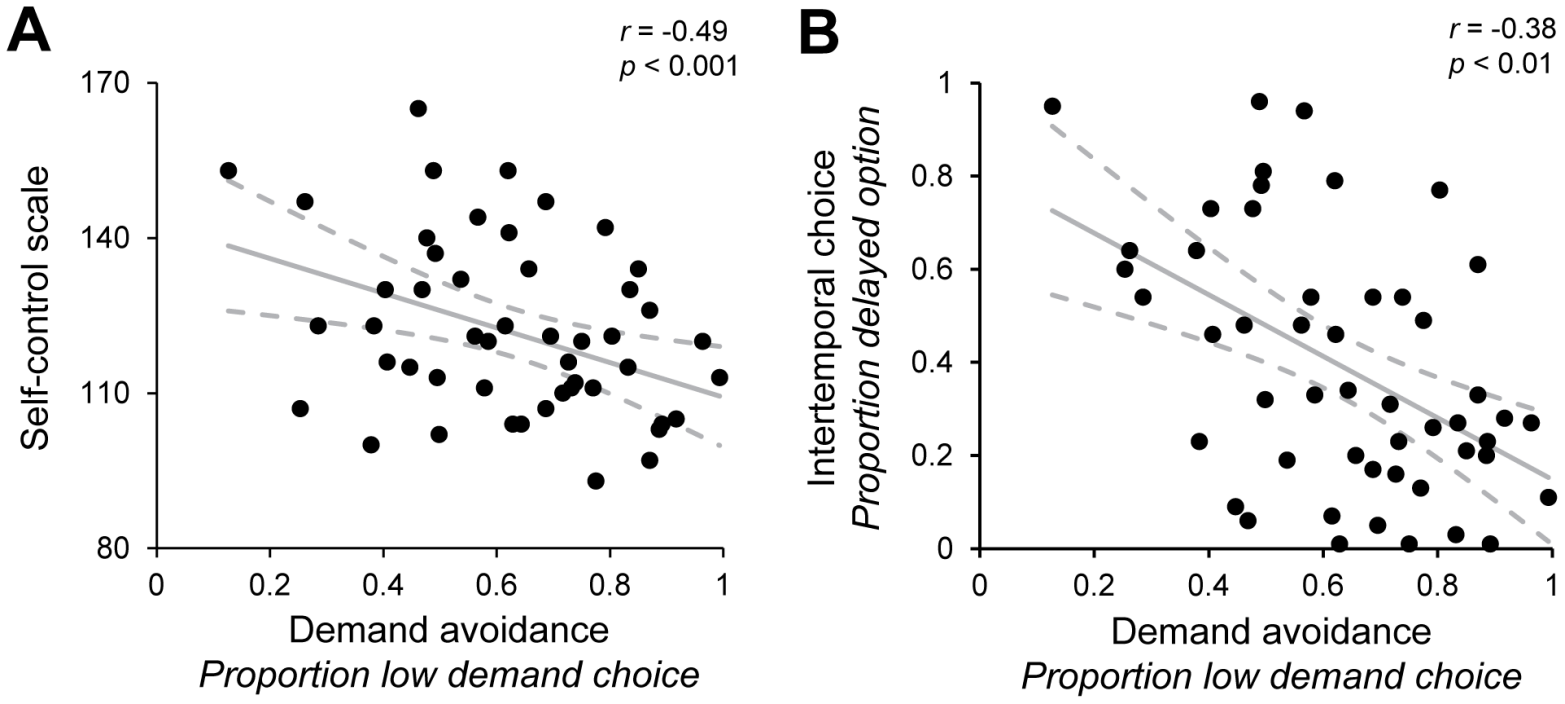
Results of individual differences experiment. (A) Relationship between demand avoidance in the DST and Self-Control Scale score. Each point corresponds to a single participant. (B) Relationship between demand avoidance in the DST and the proportion of delayed-option choices in the ITC task.

Consistent with earlier studies, participants as a group displayed an overall demand avoidance effect in the DST, tending to favor the low-demand option, *t*(49) = 4.60, *p*<0.0001, but also showed considerable cross-participant variability (see Figure 3A–B). As predicted, this variability tracked individual differences on the two self-control measures. First, demand avoidance on the DST correlated negatively with scores on the Self-Control Scale, with a higher proportion of low-demand choices in the DST predicting a lower quantity of self-regulatory behavior reported in the Self-Control Scale (*r*(48) = −.38, *p*<0.01; Figure 3A). Second, DST performance correlated with choice behavior in the ITC task, with a greater proportion of low-demand choices in the DST predicting a smaller proportion of delayed-option ITC choices (*r*(48) = −0.49, *p*<0.001; Figure 3B).

**Figure 4:**
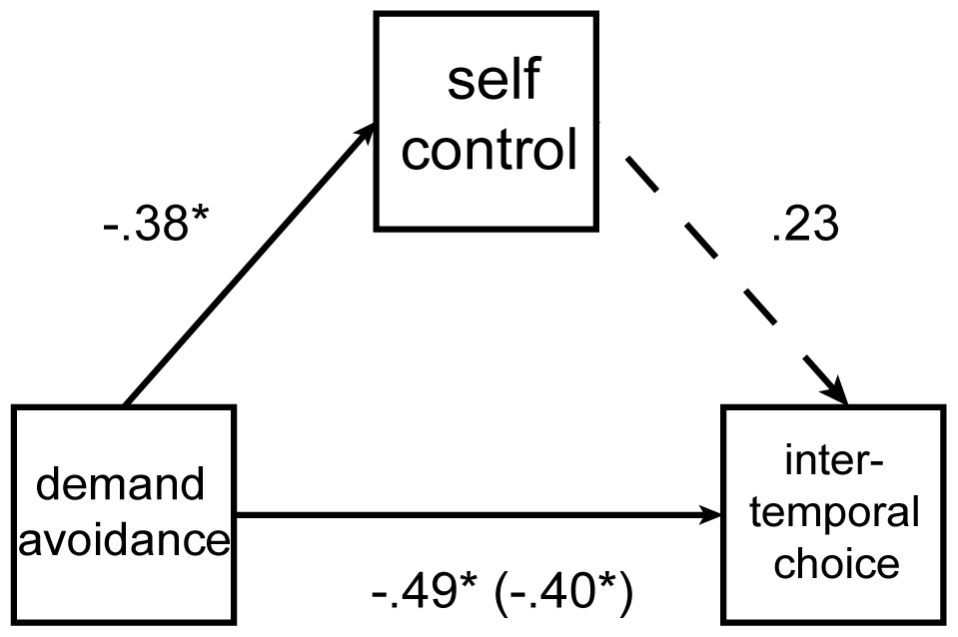
Results from a mediation analysis. This figure shows the relationship between demand avoidance and ITC, as mediated by the Self-Control Scale measure (“self-control”). Numeric labels indicate standardized regression coefficients deriving from an analysis regressing self-control onto demand avoidance (upper left), an analysis regressing ITC onto demand avoidance (coefficient in parentheses), and an analysis regressing ITC onto both self-control (upper right) and demand avoidance (bottom). Demand avoidance explains variance in both ITC and in Self-Control Scale scores. Although Self-Control Scale score predicts ITC, this effect falls below statistical significance when demand avoidance is included as an additional regressor. Thus, demand avoidance appears to reflect a common factor underlying both Self-Control Scale responses and ITC behavior.

Results indicated a positive correlation between our two measures of self-control: Participants with high point-scores on the Self-Control Scale also tended more often to select the delayed option in the ITC task (*r*(48) = 0.39, *p*<0.01). However, a mediation analysis indicated that DST performance continued to predict each self-control measure even after the other self-control measure was covaried out (Figure 4). Factoring out the relationship between each self-control measure and demand avoidance reduced the correlation between the two self-control measures below the threshold for statistical significance (see Figure 4), suggesting that the DST tapped a factor common to both.

## Discussion

In sum, the results of this individual-differences study confirmed an inverse relationship between cognitive demand avoidance and the efficacy of self-control. Together with the finding that cognitive effort costs and self-control relate to common areas within dlPFC, this result lends considerable support to the idea that the exertion of self-control carries intrinsic subjective costs.

As discussed in the Introduction, the cost of self-control plays a pivotal role in the influential dual-self model that has emerged from economics, empowering that model to account for a wide range of behavioral effects [5]–[7]. The present findings bolster the psychological plausibility of the dual-self model, providing empirical confirmation for one of its key stipulations, that self-control carries an intrinsic cost.

The precise characterization of control costs has in fact taken two subtly different forms in economic dual-self models. In some models, a cost attaches directly to the exertion of top-down control [5]; others frame the cost of control as an opportunity cost, arising when self-control requires the short-term ‘self’ to forego tempting immediate reward [25]. These two possibilities are difficult to differentiate empirically, since control demands will generally increase with temptation [17]. However, the present results offer differential support for the idea that self-control exertion carries an inherent cost, since this view (but not the opportunity-cost alternative) provides an explanation for why self-control should correlate with demand avoidance in the DST.

By validating the notion of self-control costs, our findings also indirectly support the other key tenet of the economic model, the idea that choice is governed by two ‘selves’ with differing preferences, and that self-control reflects the ascendency of one of these selves – the one with more patient preferences – over the other^3^. This notion, and the dual modes of valuation that it implies, is not universal among formal models of self-control. Indeed, theories involving a single, fixed utility function remain widely considered^4^, especially in work on ITC [14], [15]. However, in contrast to the dual-self framework, such a perspective provides no obvious entrypoint for effort costs, since it includes no distinct self-control function to which such costs might attach.

In addition to its longstanding role in economic models, the notion of self-control costs has very recently begun to appear in psychological theories of self-control failure and ‘ego depletion.’ For many years, work in this area has been dominated by the idea that self-control draws on a limited resource – possibly glucose [26], [27] – and that impulsive behavior arises when this resource is depleted, making the exertion of self-control impossible [28], [29]. Over time, however, accumulating empirical observations have placed an increasing strain on the resource account [30]–[32], contributing to an emerging trend toward motivation-based theories of self-control failure. Under this emerging perspective, self-control failures arise not from an inability to self-regulate, but from a *decision* not to do so, based on a cost-benefit analysis that takes into account the intrinsic cost of self-control [31], [32]. The present results provide additional encouragement for this reformulation, inviting further research into the details and dynamics of the relevant cost-benefit analyses [33].

Together with its implications for psychological and economic models, the present findings add to recent neuroscientific evidence implicating the dlPFC in self-control and ITC. Despite the relevant findings reviewed earlier, some important negative results have left room for uncertainty, especially in the case of ITC [14], [15]. Our results indirectly support the relevance of dlPFC, by providing evidence that self-control and ITC are associated with effort costs, costs that the dlPFC has been shown to index [21]. At a broader level, the present findings establish a new bridge between neuroscientific research on self-control and parallel research on effort costs and demand avoidance, prompting further investigations into the relationship between these two domains.

For example, future work could employ fMRI or transcranial magnetic stimulation methods to more directly test for the role of dlPFC in representing effort costs during self-control. One possibility would be to measure individual differences in dlPFC sensitivity to cognitive effort and predict individual differences in behavior and prefrontal activity during self-control (and vice versa). In addition, the current results suggest that other forms of decision making that depend on activity in dlPFC may show similar sensitivities to individual differences in effort costs. For example, one might predict that an aversion to cognitive effort predicts less utilitarian moral reasoning [34] and increased reliance on habit or model-free reinforcement learning [35], since these cognitive functions are dependent on computation implemented by the dlPFC.

### Notes

1. This effect appeared in McClure et al. [9] as a statistical trend. Nevertheless, that paper concluded that activity within a network including the dlPFC “is associated with choice, such that lesser activity…predict[s] a greater likelihood of choosing the sooner, lesser option” (p. 5801).
2. According to one view, stemming from McClure et al. [10] the dlPFC participates in one of two competing systems, each of which carries its own representation of choice value. Under a contrasting account stemming from Hare et al. [8], the brain carries only a single representation of value, but one that is subject to top-down modulation by the dlPFC. Despite the important differences between these theories as accounts of neural implementation, it is important to note that they are both entirely consistent with the more abstract dual-self framework. Under both neuroscientific theories, self-control depends upon the activity of a distinct mechanism, which overrides the behavioral preferences arising from a second, more basic, system. This scenario aligns precisely with the dual-self model, regardless of whether the override operation occurs through competition or through modulation.
3. As explained in Note 2, this idea is equally consistent with neuroscientific accounts positing direct competition between independent value representations [10], or top-down modulation of a single representation of value [8].
4. The single-utility view is commonly attributed to Kable and Glimcher [14], [15], and their proposals can be so interpreted. However, as Hare and colleagues [8] noted, the model advanced by Kable and Glimcher [15] does not explicitly rule out top-down modulation of value representations. In fact, Kable and Glimcher [14] explicitly left open the possibility that top-down modulation, perhaps driven by dlPFC, might play a role. Some caution is thus required in framing the debate.

## Supporting Information

Information S1 (DOCX)
