# Supplementary material for: Neural and Behavioral Evidence for an Intrinsic Cost of Self-Control
Source: PLoS One. 2013 Aug 27;8(8):e72626. doi: 10.1371/journal.pone.0072626 (PMC3754929; doi:10.1371/journal.pone.0072626)
Supplement: Information S1 — (DOCX) [file pone.0072626.s001.docx]

Supporting Information

Demonstration of effort-discounting in dlPFC

In the fMRI study on ‘effort discounting’ by Botvinick, Huffstettler, and McGuire [1], described in detail in the original publication, participants completed task blocks with either low or high demands for task switching. After each block, participants either received or did not receive a reward presented as payment for the task (independent of accuracy).

In the analysis originally reported by Botvinick and colleagues [1], single-subject general linear models (GLM) analyses estimated a trial-by-trial beta coefficient for each task block and reward delivery event. There were 72 trials in the experiment, each consisting of a task block followed by a reward. This initial analysis therefore yielded, for each participant, whole-brain maps of 72 task-block coefficients and 72 corresponding reward-event coefficients. Trials were approximately evenly distributed across the cells of a 2x2 design, crossing task demand (high vs. low) with reward magnitude (high vs. low).

For the central analysis of the previous report [1], these single-event coefficients were spatially averaged in two anatomical regions of interest (ROIs), located in anterior cingulate cortex (ACC) and nucleus accumbens (NAc). A regression model then tested the lagged functional connectivity between ACC (in the task phase) and NAc (in the subsequent reward phase) across trials. The reward-phase NAc response was the outcome variable in the regression model, and there were four predictors: demand level, reward magnitude, task-phase ACC response, and task-phase NAc response. (Task-phase NAc response was included to account for baseline fluctuations.) The key finding was that, even controlling for the other predictors, task-phase activity in ACC was negatively related to subsequent reward-phase activity in NAc. These results suggested an effort discounting effect: an increase in cognitive costs, as registered by the ACC, decreased the reward response in the NAc.

The objective of the present reanalysis was to test, in a voxelwise manner across the entire brain, whether other areas would show the same effect that this analysis detected in ACC. Our interest was particularly centered on dlPFC. In our original study [1], we reported a region-of-interest (ROI) analysis within this region, which failed to reveal the effect of interest, a finding that led us to conclude that dlPFC is not involved in the registration of effort costs. However, a subsequent study [2] found that activity in *both* ACC and a region of dlPFC correlated with demand avoidance. Importantly, the dlPFC area identified in this study differed slightly from the region targeted in the ROI analysis in Botvinick et al. [1]. Based on this, we speculated that the present whole-brain approach might reveal a dlPFC effect in the Botvinick et al. [1] dataset.

The new analysis tests the relationship between task-phase activity in each voxel and the subsequent reward response in the NAc ROI. In other words, we tested each voxel's lagged functional connectivity, across trials, with a seed region in NAc.

The terms in the regression model were rearranged to facilitate whole-brain exploratory analysis, but their relationships remained the same. Whole-brain maps of task-phase beta coefficients (72 per participant, each corresponding to one trial) served as the outcome data (see the original report for details of the estimation of these trial-wise coefficients). The predictor of interest was the reward-phase NAc response. Additional predictors were demand level, reward magnitude, and task-phase NAc response. A voxelwise regression analysis for each participant was conducted using AFNI software [3]. The relationship between each voxel's task-phase response and the reward-phase NAc response (controlling for the other three variables) served as an index of the effort discounting effect at that location. A permutation test (the FSL function “Randomise”) was used to produce a whole-brain map of clusters (controlled for family-wise error rate) that correlated with the effort discounting effect. Pertinent to the current study, this new analysis revealed an area in bilateral dlPFC (depicted in Figure 1 of the main text) that negatively correlated with reward response in the NAc, implicating a role for this area in effort discounting.

A comment is warranted given the relationship between this dlPFC effect, which provides the focus of the present work, and the ACC effect originally reported. In McGuire et al. [2], we offered some speculations concerning potential detailed differences between the roles played by dlPFC and ACC in effort-cost evaluation. While such fine-grained differences remain tenable, it should be noted that they are not directly relevant to the hypotheses at stake in the present work. The key assumption, for present purposes, is simply that dlPFC engagement is relevant to effort-cost registration and avoidance.

Neuroimaging meta-analysis

To compare the regions of dlPFC recruited during self-control and the representation of cognitive costs, we leveraged data from five published fMRI studies, two focusing on effort cost representations and three on self-control.

The first neuroimaging dataset on cognitive costs came from the effort discounting study reported above. In the second study, performed by McGuire and Botvinick [2], participants completed an version of the DST in which participants performed a blocks of task-switching trials, rating after each the degree to which they wished to avoid similar task blocks in future. As described in detail in the original report, these avoidance ratings were correlated with the degree to which the dlPFC was recruited during task performance, even after controlling for reaction times and error rates.

The experiments self-control comprised of two intertemporal choice studies performed by McClure and colleagues [4,5] and one study on self-control in self-described dieters by Hare, Camerer and Rangel [6].

In the intertemporal choice experiments, participants performed an ITC task similar to the one described above in conjunction with our behavioral study. In one experiment, participants chose between Amazon gifts card with different dollar values and delays, whereas in the other, participants the rewards were squirts of juice or water administered during the scanning session. As described in the original reports, the authors discovered a network of brain regions, including dlPFC, which increased activation when the delayed larger reward was selected (see Figure 1 of main text).

Hare and colleagues [6] presented dieters with choices between healthy and unhealthy foods. They found that when participants showed failures in self-control, i.e., when they selected the unhealthy option, activity in a region of the dlPFC (see Figure 1 in main text) decreased. Bolstering the idea that this region was implicated in self-control, task-related fluctuations in this region were functionally related with goal values represented in the ventromedial PFC.

As a first step in our analyses, we created functional masks based on the dlPFC regions identified in each of the five studies. Next, we used AFNI’s 3dAllineate function [3] to warp the datasets that were originally in Montreal Neurological Institute (MNI; all datasets, except for the McGuire and Botvinick demand avoidance experiment) space to Talairach space. The resulting dlPFC masks are the ones displayed in Figure 1 of the main text.

In order to perform our region-of-interest (ROI) analyses, we took the whole-brain maps of correlations between task-phase activity and subsequent avoidance ratings in the McGuire and Botvinick [2] demand avoidance study (*n* = 10) and of correlations between task-phase activity and the subsequent reward-related response in the NAc in the Botvinick and colleagues [1] effort discounting study (*n* = 23). For each participant, we computed the average correlation in each of the three dlPFC clusters from the self-control datasets (Talaraich-transformed for the McGuire and Botvinick dataset). For both studies and all three dlPFC clusters, group-level t-tests revealed that the task-phase signal showed a significant correlation with avoidance ratings (Table 1, top row) and activity in the NAc (Table 1, bottom row).

References

1. Botvinick MM, Huffstetler S, McGuire JT (2009) Effort discounting in human nucleus accumbens. Cogn Affect Behav Neurosci 9: 16-27.

2. McGuire JT, Botvinick MM (2010) Prefrontal cortex, cognitive control, and the registration of decision costs. Proc Nat Acad Sci USA 107: 7922–7926.

3. Cox R (1996) AFNI: software for analysis and visualization of functional magnetic resonance neuroimages. Comput Biomed Res 29: 162-173.

4. McClure SM, Ericson KM, Laibson DI, Loewenstein G, Cohen JD (2007) Time discounting for primary rewards. J Neurosci 27: 5796.

5. McClure SM, Laibson D, Loewenstein G, Cohen JD (2004) Separate neural systems value immediate and delayed monetary rewards. Science 306: 503-507.

6. Hare TA, Camerer CF, Rangel A (2009) Self-control in decision-making involves modulation of the vmPFC valuation system. Science 324: 646-648.
